# Exploring the impact of magnetic fields on biomass production efficiency under aerobic and anaerobic batch fermentation of Saccharomyces cerevisiae

**DOI:** 10.1038/s41598-024-63628-1

**Published:** 2024-06-04

**Authors:** M. Sincak, M. Turker, Ü. C. Derman, A. Erdem, P. Jandacka, M. Luptak, A. Luptakova, J. Sedlakova-Kadukova

**Affiliations:** 1 Faculty of Natural Science, University of Ss. Cyril and Methodius in Trnava, Nam. J. Herdu 2, 917 01 Trnava, Slovakia; 2 Pak Gida Uretim Ve Paz. A.S., Kartepe, Kocaeli Turkey; 3 Faculty of Forestry and Wood Sciences, Czech University of Life Sciences Prague, Kamycka 129, 16500 Praha 6 – Suchdol, Czech Republic; 4 Faculty of Materials, Metallurgy and Recycling, Technical University of Kosice, Letna 9, 04200 Kosice, Slovakia; 5 Institute of Geotechnics, Slovak Academy of Sciences, Watsonova 45, 04001 Kosice, Slovakia; 6 ALGAJAS s.r.o., Prazská 16, 04011 Kosice, Slovakia

**Keywords:** Magnetic field, Yeast, Batch fermentation, Aerobic, Anaerobic, Biomass, Metabolism acceleration, Biological techniques, Biotechnology, Microbiology

## Abstract

In this work, the effect of moderate electromagnetic fields (2.5, 10, and 15 mT) was studied using an immersed coil inserted directly into a bioreactor on batch cultivation of yeast under both aerobic and anaerobic conditions. Throughout the cultivation, parameters, including CO_2_ levels, O_2_ saturation, nitrogen consumption, glucose uptake, ethanol production, and yeast growth (using OD 600 measurements at 1-h intervals), were analysed. The results showed that 10 and 15 mT magnetic fields not only statistically significantly boosted and sped up biomass production (by 38–70%), but also accelerated overall metabolism, accelerating glucose, oxygen, and nitrogen consumption, by 1–2 h. The carbon balance analysis revealed an acceleration in ethanol and glycerol production, albeit with final concentrations by 22–28% lower, with a more pronounced effect in aerobic cultivation. These findings suggest that magnetic fields shift the metabolic balance toward biomass formation rather than ethanol production, showcasing their potential to modulate yeast metabolism. Considering coil heating, opting for the 10 mT magnetic field is preferable due to its lower heat generation. In these terms, we propose that magnetic field can be used as novel tool to increase biomass yield and accelerate yeast metabolism.

## Introduction

Although the utilization of living organisms in industry is increasingly common, effectively controlling biotechnological processes remains a significant challenge. Key hurdles include contamination control, precise management of temperature and pH, regulation of oxygen levels, ensuring nutrient availability, and selecting strains with optimal properties. Addressing these challenges requires ongoing research to explore alternative methods for enhancing biotechnological processes. Traditional process control methods involve rigorous implementation of aseptic techniques, sterile equipment and environments, continuous monitoring and adjustment of various parameters like temperature, pH, oxygen levels, and nutrient concentrations in fermentation media to promote robust growth and metabolic activity^1^. Therefore, the study and implementation of new strategies for controlling biotechnological processes are essential to ensure desired reaction progression, high yield, and cost minimization^2^.

In recent years, the potential of utilizing magnetic fields has garnered attention as a promising avenue for biotechnological process control. Magnetic fields offer non-invasive characteristics and can serve as external environmental factors applicable across a wide spectrum of industrial biotechnological processes. Moreover, their usage presents a sustainable, environmentally friendly alternative, especially when employing magnets or weak electric fields, which have the potential to positively influence diverse biotechnological processes ^3^. This positions magnetic fields as competitive alternatives to traditional, less ecologically friendly methods^4,5^.

Given their non-invasive nature, magnetic fields hold considerable promise across various biotechnological applications by preserving the characteristics and quality of the medium. Despite acknowledging the potential of magnetic fields in influencing cellular processes, there remains a notable scarcity of practical applications in this area, necessitating further exploration in future studies^3^.

Yeast fermentation, particularly employing Saccharomyces cerevisiae, is integral to various industrial processes, serving as the biological engine behind the production of a wide array of products, including bread, alcoholic beverages, biofuels, and pharmaceuticals^6^. While yeast fermentation offers significant advantages such as high efficiency, scalability, and cost-effectiveness, it also presents challenges like susceptibility to contamination, the need for precise environmental control, and the requirement to optimize yields while minimizing byproducts and energy consumption^7^.

The impact of electromagnetic fields on yeast growth, metabolism, and ethanol production is a complex phenomenon that has been studied by a limited number of scientists, with varying results. While some researchers observed positive effects of magnetic fields on yeast fermentation, others reported negative impacts or found no significant effects^8–12^. Notably, one study conducted on a larger scale (4 L) reported positive effects of electromagnetic fields on yeast fermentation, with an increase in ethanol production and a reduction in fermentation time^13^. However, the static magnetic field used in this study differs from the electromagnetic field induced by a direct current electric field in our research.

It is crucial to recognize that the effects of electromagnetic fields on yeast are influenced by factors such as field strength, frequency, exposure time, and the specific biological state of the cells. These variables interact in complex ways, resulting in both stimulatory and inhibitory effects on yeast metabolism and fermentation^14^. Therefore, our study represents the first attempt to control and enhance yeast-based biotechnological processes using weak static magnetic fields with magnetic flux densities (B) of 2.5, 10, and 15 mT generated by an immersed electromagnetic coil. Unlike previous research primarily focused on small-scale applications, our study extends its scope to include fermenters with a working volume of 2 L. We believe that integrating these weak electromagnetic fields into industrial settings could significantly enhance conventional processes by expediting specific yeast metabolic processes without altering the essential characteristics of the cultivation media or the final product. Furthermore, the minimal energy input required for generating these weak magnetic fields makes them a cost-effective option for integration into industrial biotechnological process control systems.

## Results and discussion

### Aerobic cultivation

#### Biomass production

**Figure 1:**
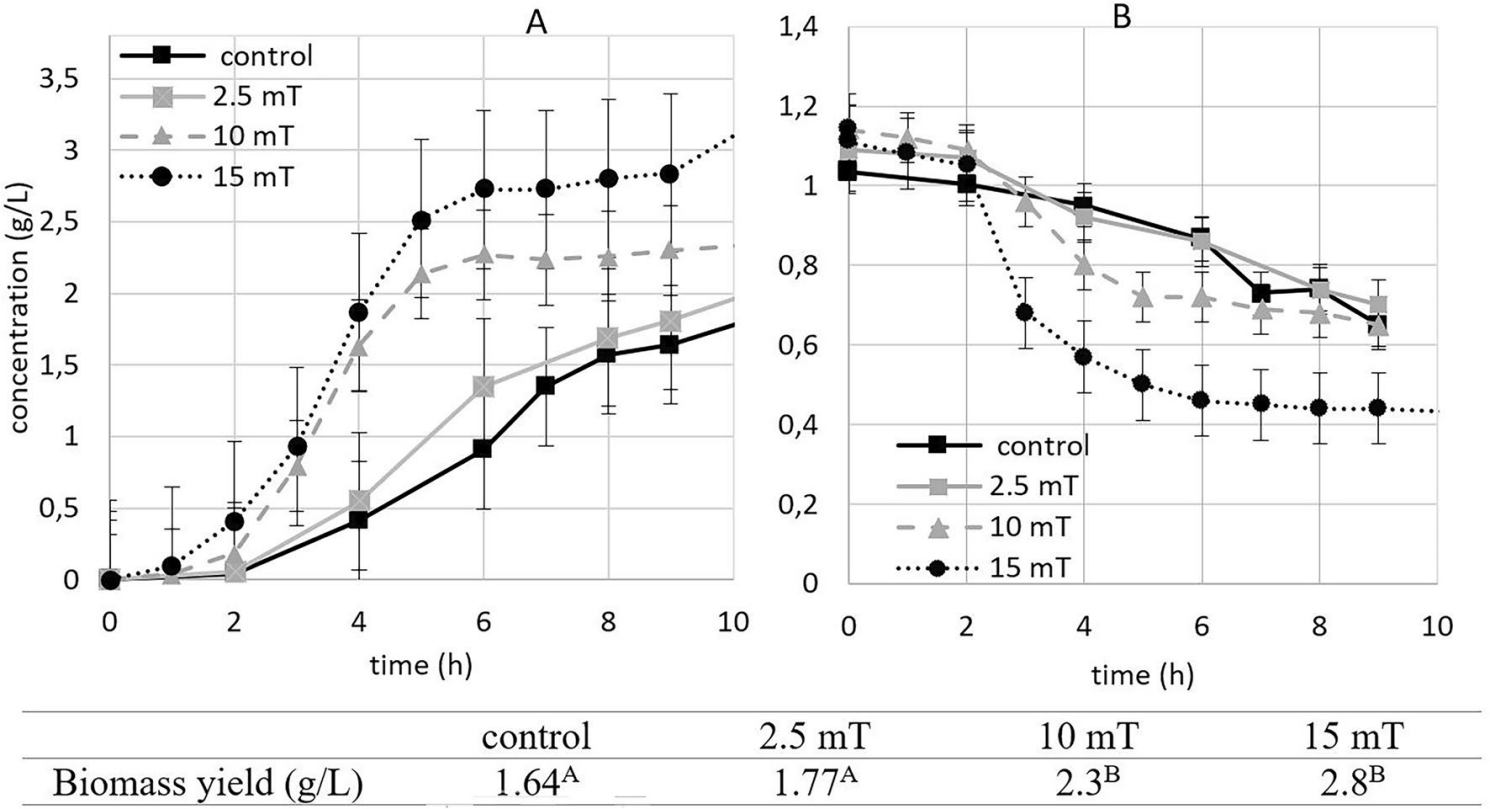
The effect of electromagnetic fields (2.5, 10 and 15 mT) on biomass production (**A**) and ammonium nitrogen consumption (**B**) under aerobic (experimental parameters: mixing: 500 RPM, aeration with oxygen/nitrogen: 2000 cm^3^/min, pH: 5.5, temperature: 30 °C). The Groups A and B are statistically significantly different according to linear regression model test (*statistically significant at *p* ≥ 0.05).

During the aerobic experiments with magnetic fields of 2.5, 10 and 15 mT, (Fig. 1) we observed a statistically significant increase in biomass yield only in case of 10 and 15 mT. The biomass production yield was 1.4 times higher using 10 mT, while with 15 mT, was it 1.7 time higher compared to control after 9 h of cultivation. A magnetic field of 2.5 mT was not strong enough to significantly impact yeast growth or metabolism.

The results of the regression analysis indicate a statistically significant increase of biomass production under magnetic fields of 10 and 15 mT by 50 and 60%, respectively, compared to both the control and 2.5 mT conditions (Supplementary Figure S1).

In addition to the increase in total biomass, we observed an acceleration of the growth rate by approximately 2 h when yeasts were exposed to magnetic fields of 10 and 15 mT. The increase in biomass concentration was also justified by decrease in ammonia concentrations in the medium as shown in Fig. 1B. The decrease in nitrogen content serves as an indirect indicator of biomass growth since all the nitrogen present in the medium is incorporated into the biomass^15^. This further confirm higher biomass yield in the case of 10 and 15 mT electromagnetic fields exposure.

#### Ethanol production

**Figure 2:**
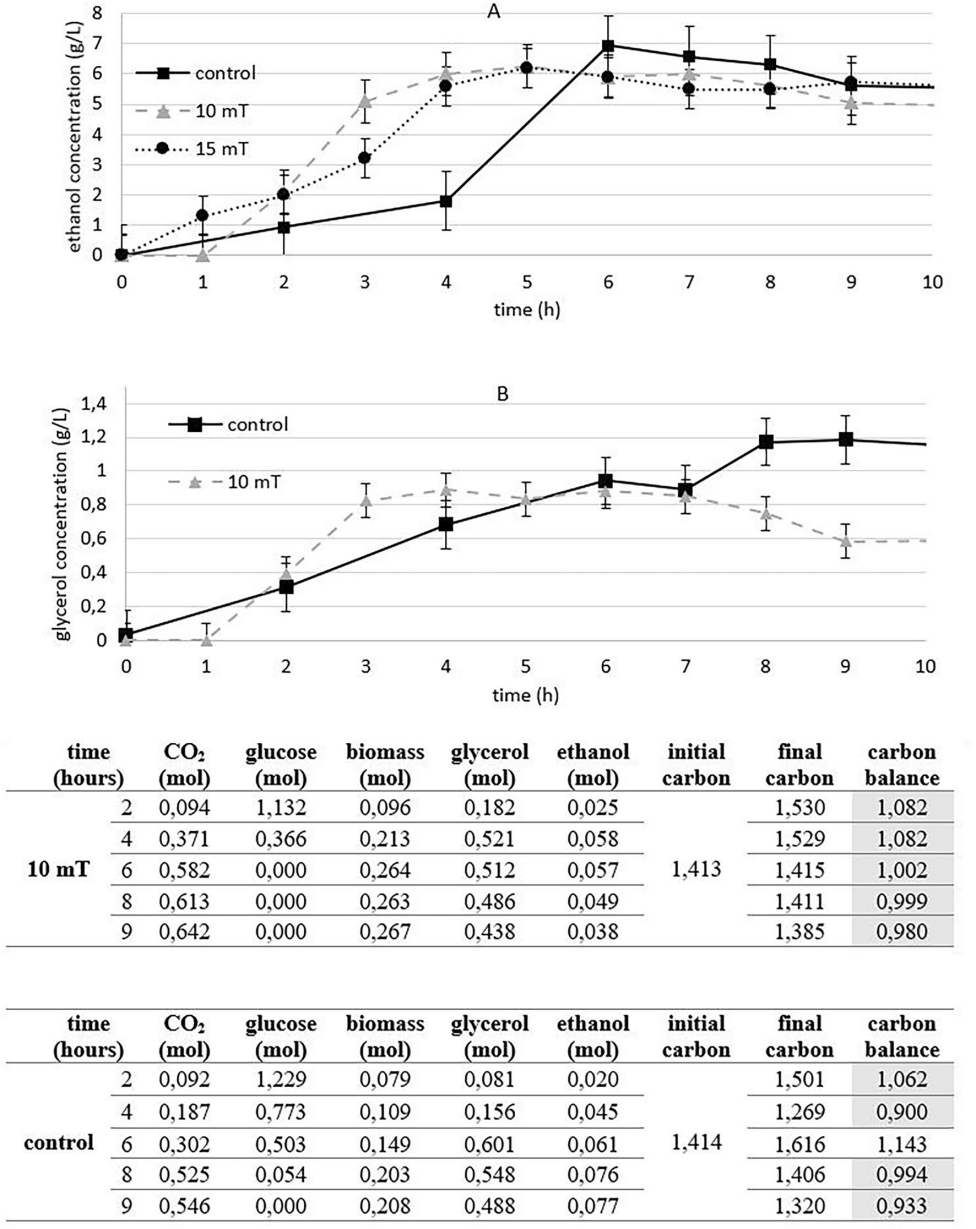
(**A**) The effect of electromagnetic fields (10 and 15 mT) on ethanol production under aerobic conditions and (**B**) The effect of electromagnetic fields (10 mT) on glycerol production under aerobic conditions with carbon balance calculations (experimental parameters: mixing: 500 RPM, aeration with oxygen/nitrogen: 2000 cm^3^/min, pH: 5.5, temperature: 30 °C), shaded cell indicated the carbon balance of 1 + /− 0.1.

In addition to its impact on biomass production, the magnetic field also influenced ethanol production. The experimental cultures exposed to magnetic fields of 10 and 15 mT exhibited faster ethanol production compared to the control. While the reduction in ethanol and glycerol production may not be immediately evident in the results obtained by HPLC, it should be considered based on the carbon balance calculation (Fig. 2A and Supplementary Figure S2). For both the experimental culture exposed to a magnetic field of 10 mT and the control culture, we calculated a carbon balance of 1 + /− 0.1. A carbon balance of 1 + /− 0.1 indicates that the amount of carbon consumed by the yeast during fermentation is equal to the amount of carbon produced as metabolic products^16^ and denotes experimental data accuracy.

Similar to ethanol production, the production of glycerol was also affected by a 10 mT magnetic field. In case of glycerol, we observed 50% reduction in glycerol production after 9 h of cultivation (Fig. 2B).

The glycerol production is related to ethanol production. Glycerol plays a crucial role during fermentation as it helps maintain osmotic and redox balance within the yeast cells. Additionally, glycerol production plays important role in NAD^+^ regeneration from NADH, which is generated during ethanol fermentation. The regeneration of NAD^+^ is essential for the continuation of fermentation processes. As glycerol yield increases, the production of NAD^+^ increases as well, leading to a decrease in ethanol yield^17^.

#### Metabolism

**Figure 3:**
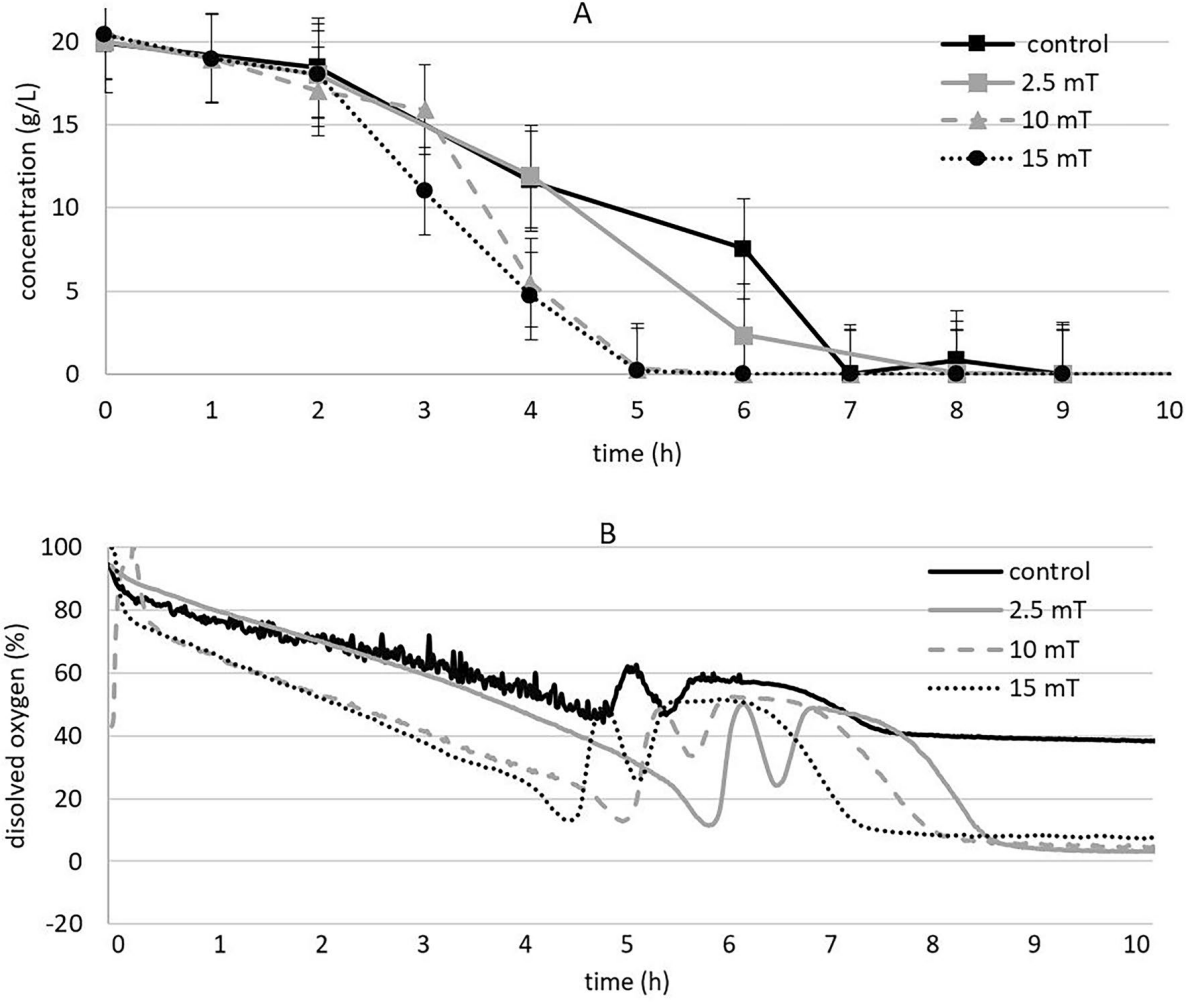
The effect of electromagnetic fields (2.5, 10 and 15 mT) on glucose consumptions (**A**) and dissolved oxygen (**B**) under aerobic conditions (experimental parameters: mixing: 500 RPM, aeration with oxygen/nitrogen: 2000 cm^3^/min, pH: 5.5, temperature: 30 °C).

In both biomass and ethanol production, we observed that the magnetic field also had an impact on the rate at which they were produced. The exposure to a magnetic field of 10 and 15 mT accelerated the rate of biomass, ethanol, and glycerol production, as well as resulted in a faster decrease in dissolved oxygen levels and glucose consumption. All of these processes were accelerated by approximately 2 h, indicating the magnetic field's ability to influence the overall metabolism of the yeast cells (Fig. 3).

**Figure 4:**
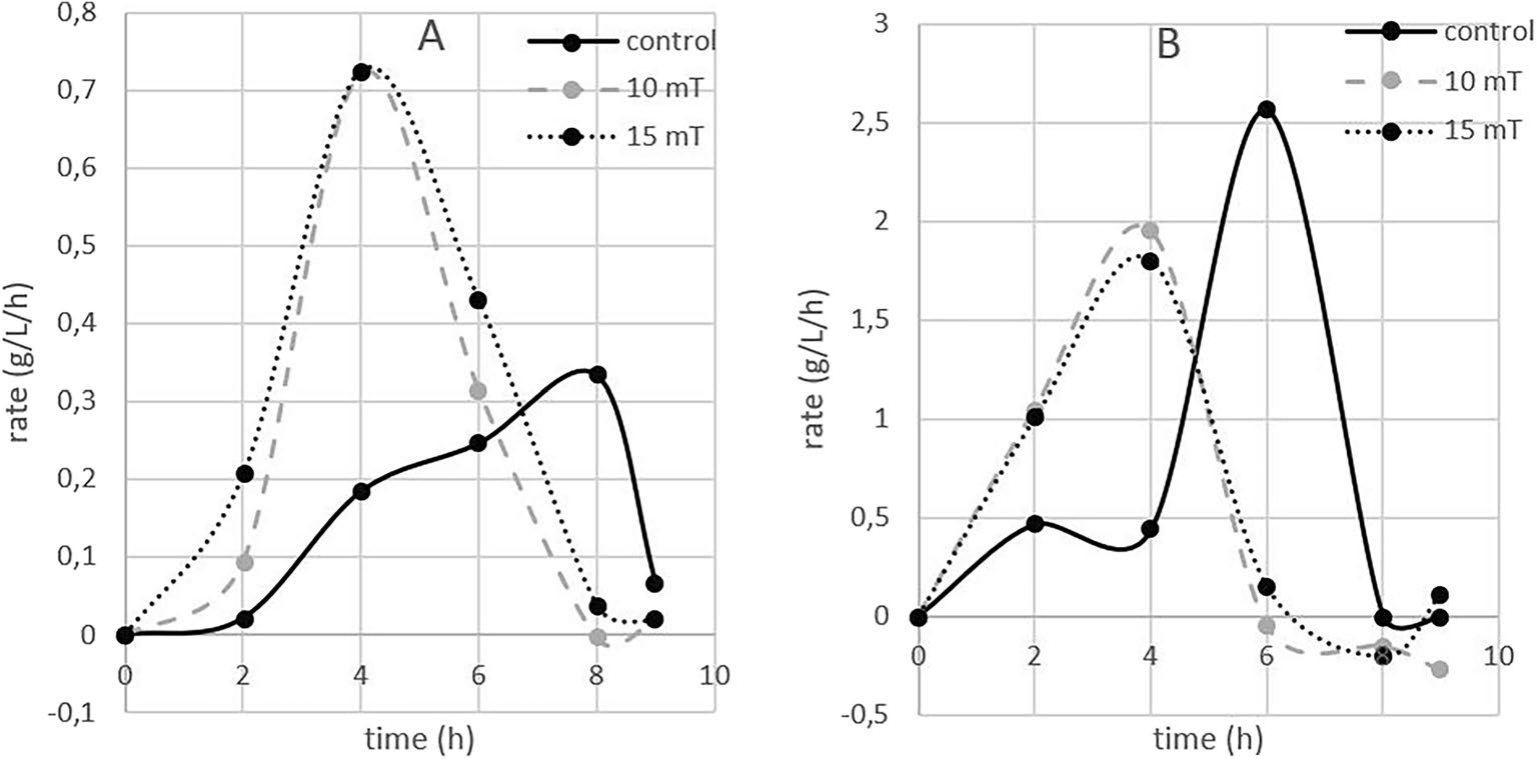
The effect of electromagnetic fields (2.5, 10 and 15 mT) on biomass rate (**A**) and ethanol rate (**B**) under aerobic conditions (experimental parameters: mixing: 500 RPM, aeration with oxygen/nitrogen: 2000 cm^3^/min, pH: 5.5, temperature: 30 °C).

As shown in Fig. 4B, we observed a fast decrease in dissolved oxygen levels as the strength (flux density) of the electromagnetic field increased, even in the case of 2.5 mT. This rapid glucose consumption and decrease in oxygen levels indicate that the yeasts are capable of more efficient utilizing the nutrients present in the medium to produce ethanol and biomass at a higher rate within a shorter time period.

When the dissolved oxygen level (dissolve oxygen) concentration reaches its lowest point, it signifies complete consumption of glucose in the medium. At this stage, the yeast undergoes a metabolic shift, adapting to consume ethanol as a substrate instead of glucose, which is no longer present in the medium ^18^. In the control experiment, this metabolic shift occurred gradually over a long period of time, spanning from the 9th hour to the 20th hour. However, in cultures exposed to magnetic fields (2.5, 10, or 15 mT), adaptation to ethanol consumption occurs within a shorter time frame, specifically within 3, 2.9, and 2.7 h at 2.5, 10, and 15 mT, respectively. The calculation of biomass and ethanol production rates showed that the yeast culture promoted a greater amount of biomass, starting from 2 to 4 h earlier, when exposed to a magnetic field of 10 or 15 mT. These results indicate that biomass and ethanol production accelerated similarly to glucose consumption in the presence of a magnetic field (Fig. 4).

**Figure 5:**
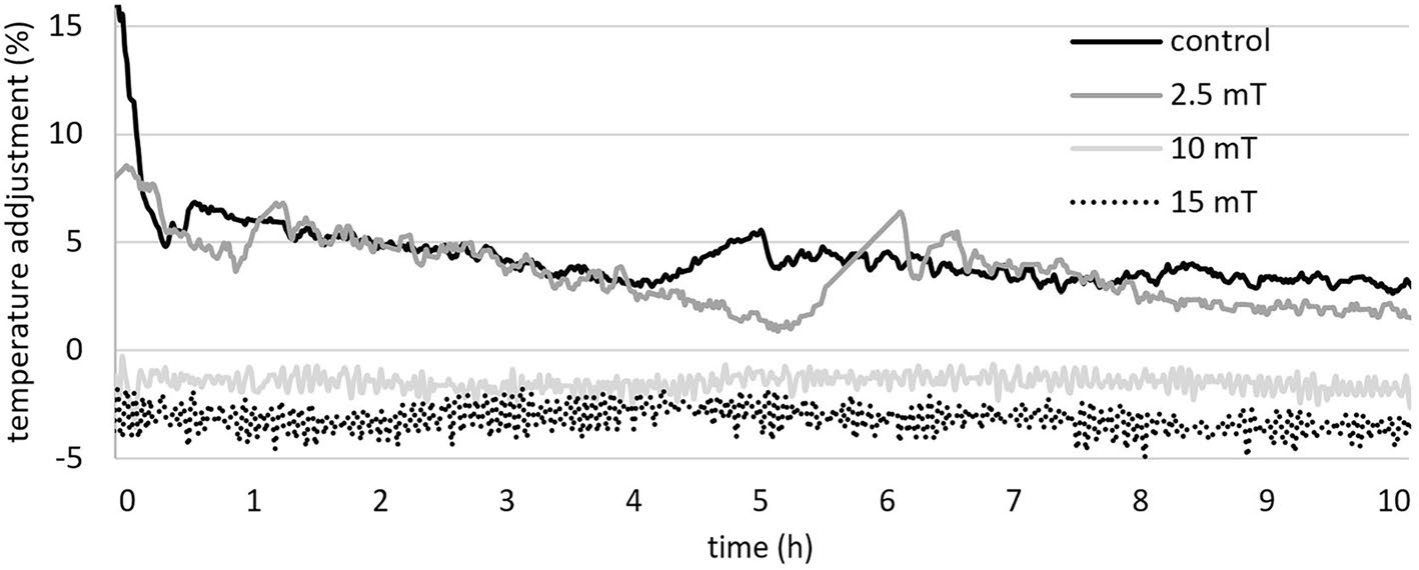
Effect of electromagnetic field (2.5, 10 and 15 mT) on the temperature in the fermenter after cooling (below zero) or heating (above zero) during the 9-h cultivation under aerobic conditions (experimental parameters: mixing: 500 RPM, aeration with oxygen/nitrogen: 2000 cm^3^/min, pH: 5.5, temperature: 30 °C).

To optimize the magnetic field parameters for industrial applications aimed at accelerating yeast growth, it is important to consider temperature as a crucial factor. Increasing the current and magnetic field strength also results in an increase in the coil temperature. In the case of a 15 mT magnetic field, the cooling system of the fermenter had to work harder to maintain a constant temperature of 30 °C (Fig. 5). This increased cooling requirement may present challenges in large-scale industrial settings.

Considering these factors, we suggest that the application of a magnetic field with a strength of 10 mT holds promise for biotechnological process control in a working volume of 2 L. This balance between magnetic field strength and temperature control can provide an optimal environment for yeast growth and metabolic acceleration in industrial applications.

**Figure 6:**
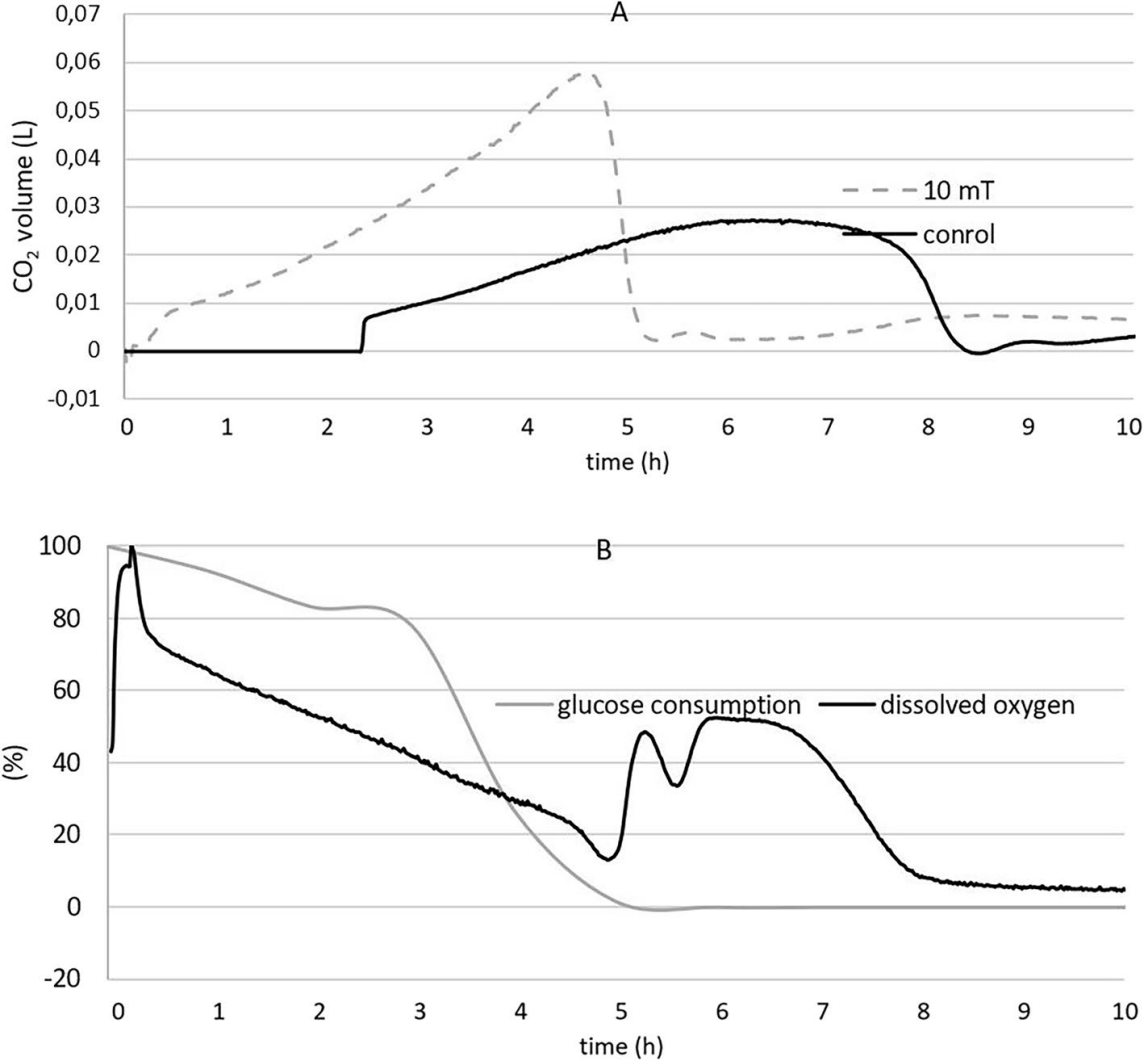
CO_2_ production after 23 h of cultivation and exposure to an electromagnetic field (10 mT) compared to that of the control (laboratory conditions – only geomagnetic field) under aerobic conditions (**A**) and the effect of electromagnetic fields (10 mT) on glucose consumption and dissolved oxygen under aerobic conditions (**B**) (experimental parameters: mixing: 500 RPM, aeration with oxygen/nitrogen: 2000 cm^3^/min, pH: 5.5, temperature: 30 °C).

Considering 10 mT as the optimal electromagnetic field for achieving comparable acceleration of growth and metabolism, we conducted several additional experiments. We observed an increase in the production of CO_2_, particularly during the initial phase of cultivation, compared to that in the control, which reached its maximum after 4.6 h of cultivation. These findings align with the depletion of glucose consumption and the decrease in dissolved oxygen in the medium, which occurred around the fifth hour of cultivation (Fig. 6).

### Anaerobic cultivation

#### Biomass production

**Figure 7:**
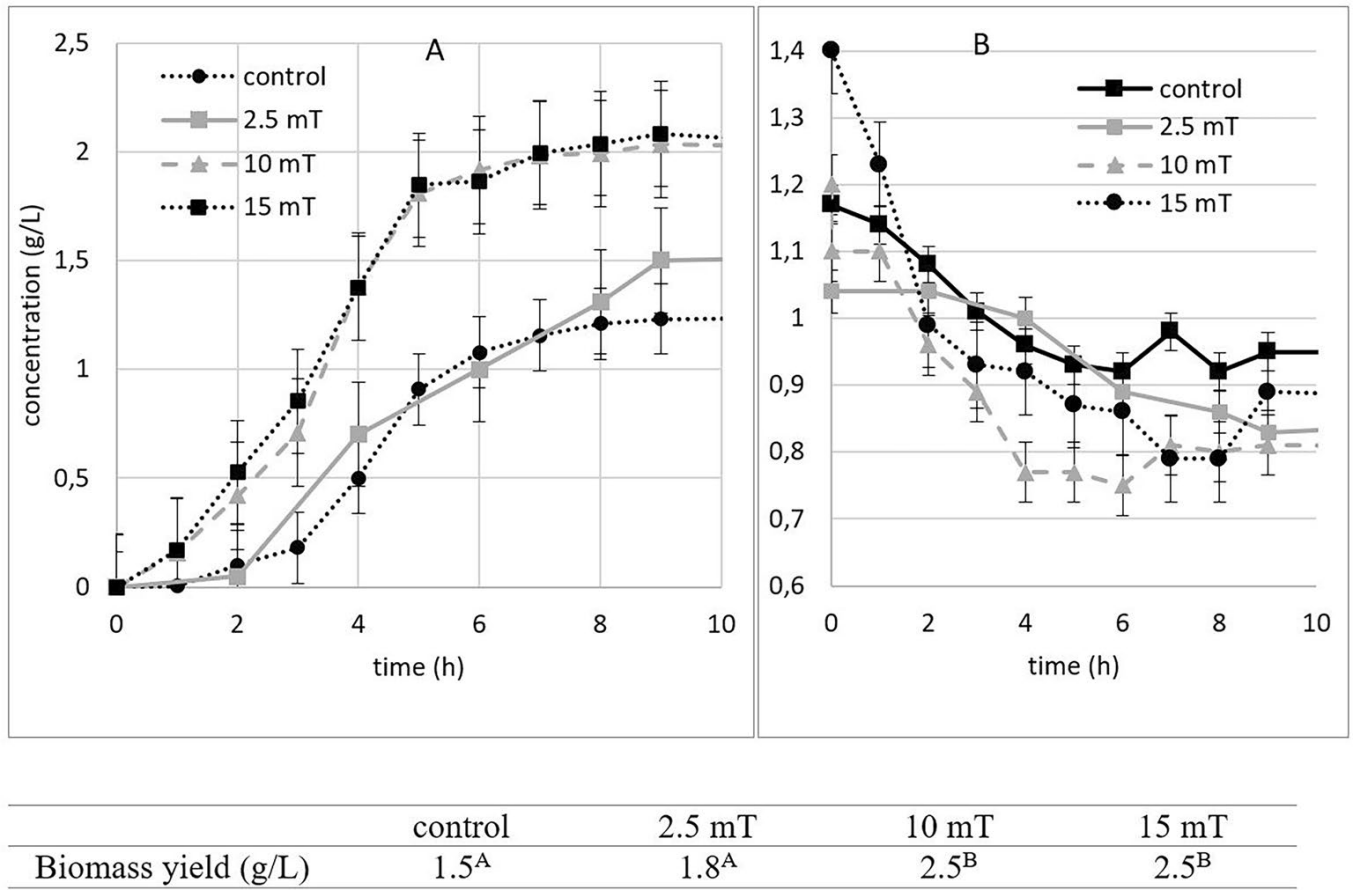
The effect of electromagnetic fields (2.5, 10 and 15 mT) on biomass production (**A**) and ammonium nitrogen consumption (**B**) under anaerobic conditions (experimental parameters: mixing: 500 RPM, aeration with oxygen/nitrogen: 2000 cm^3^/min, pH: 5.5, temperature: 30 °C). The Groups A and B are statistically significantly different according to linear regression model test (*statistically significant at *p* ≥ 0.05).

Similarly to aerobic cultivation, we observed a statistically significant increase in biomass yield using regression analyses only in the case of 10 and 15 mT (Fig. 7) during experiments with a magnetic field and magnetic flux densities of 2.5, 10 and 15 mT under anaerobic conditions, (Supplementary Figure S3).Compared to that of the control, the biomass production was 1.6 to 1.7 times higher after 9 h of cultivation. In our opinion, a magnetic field of 2.5 mT was not strong enough to significantly impact yeast growth or metabolism.

In addition to the increase in total biomass, we observed an increase in the growth rate by approximately 1 h after 10 and 15 mT magnetic field exposure. This acceleration can also be observed in the decrease in ammonium nitrogen levels, as ammonium nitrogen is incorporated into biomass (Fig. 7B). The role of nitrogen as an indirect indicator of biomass increase was described in previous section.

#### Ethanol production

**Figure 8:**
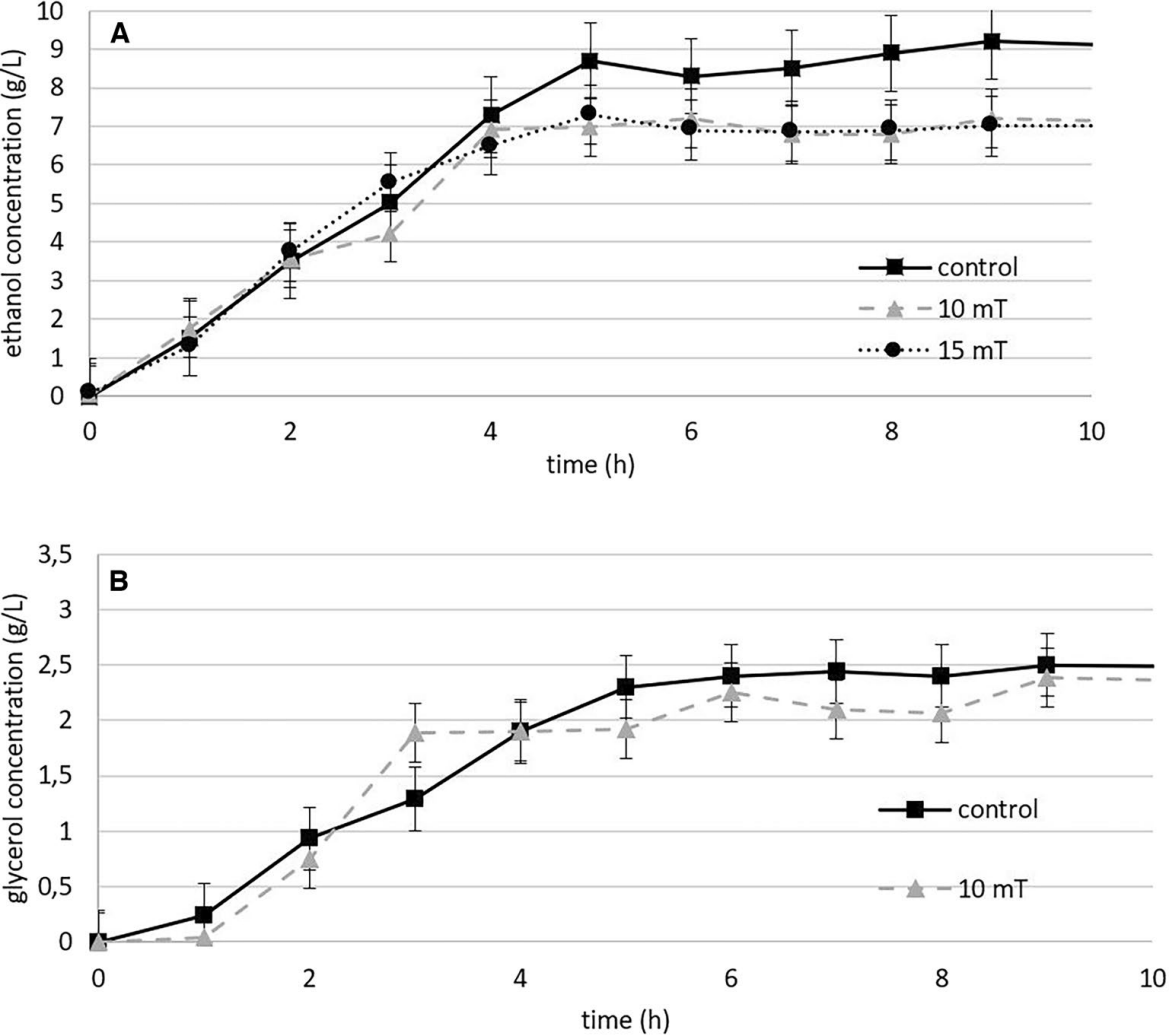
The effect of electromagnetic fields (10 and 15 mT) on ethanol production under anaerobic conditions (**A**) and the effect of electromagnetic field (10 mT) on glycerol production under anaerobic conditions (**B**) (experimental parameters: mixing: 500 RPM, aeration with oxygen/nitrogen: 2000 cm^3^/min, pH: 5.5, temperature: 30 °C).

During cultivation under anaerobic conditions, we observed that a magnetic field can increase biomass yield while leading to a decrease in ethanol yield. The overall ethanol production after 9 h of cultivation was lower in the presence of a 10 or 15 mT magnetic field (7.1 + /- 1 g/L) than in the control (9.2 g/L), representing a reduction of up to 26% although it was not statistically significant (Supplementary Figure S4). Compared to ethanol production, glycerol production was accelerated by a magnetic field of 10 mT for approximately one hour (Fig. 8). Glycerol production is closely related to ethanol production and plays important roles in fermentation, as previously described in Sect. 3.1.2.

#### Metabolism

**Figure 9:**
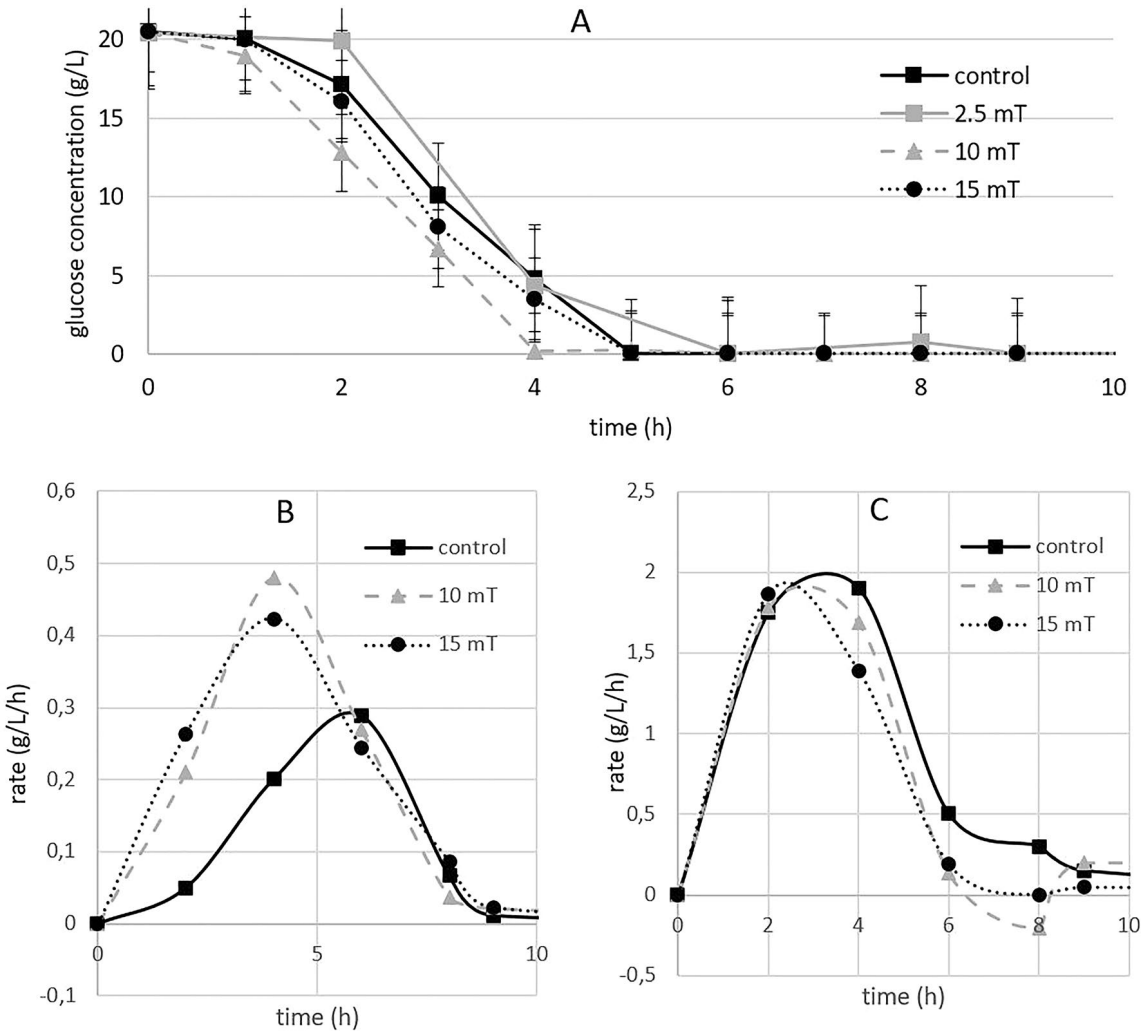
The effect of electromagnetic fields (2.5, 10 and 15 mT) on glucose consumption and (**A**) the effect of electromagnetic fields (2.5, 10 and 15 mT) on biomass production rate (**B**) and ethanol production rate (**C**) under anaerobic conditions (experimental parameters: mixing: 500 RPM, aeration with oxygen/nitrogen: 2000 cm^3^/min, pH: 5.5, temperature: 30 °C).

With respect to both biomass and ethanol production, we observed that the magnetic field influenced the final concentration of biomass and ethanol, and it also accelerated the speed of biomass and glycerol production by approximately 1 h (Fig. 9A). The calculation of the rate of biomass or ethanol production showed that the yeast culture reached the highest speed of biomass production 2 h earlier than the control, but the rate of ethanol production was not affected by the magnetic field (Fig. 9B, C).

This can be explained by the fact that in anaerobic cultivation, the conditions for ethanol production are already optimal, and after magnetic field exposure, carbon is preferably incorporated into biomass rather than being directed toward ethanol or glycerol.

**Figure 10:**
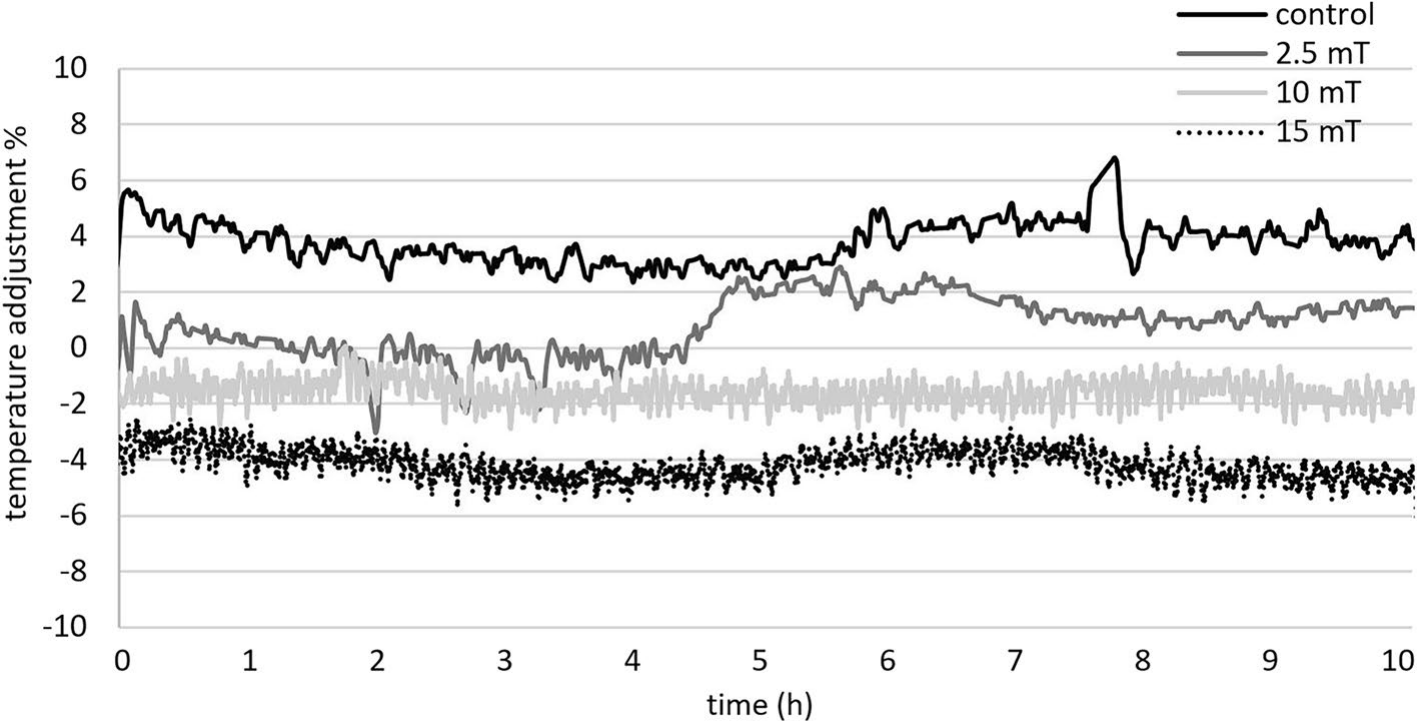
Effect of electromagnetic field (2.5, 10 and 15 mT) on the temperature in the fermenter after cooling (below zero) or heating (above zero) during the 9-h cultivation under anaerobic conditions (experimental parameters: mixing: 500 RPM, aeration with oxygen/nitrogen: 2000 cm^3^/min, pH: 5.5, temperature: 30 °C).

Similarly, as during aerobic cultivation the cooling system of the fermenter was pressed to maintain constant temperature under 15 mT magnetic field (Fig. 10) so 10 mT having similar effects was more suitable for yeast cultivation in a 2 L fermenter especially when considering large-scale industrial setting.

### Comparison of aerobic and anaerobic cultivation methods

In the case of both aerobic and anaerobic cultivation, magnetic field exposure influenced yeast metabolism, resulting in decreased ethanol or glycerol yields (for 10 or/and 15 mT magnetic fields) and a corresponding increase in biomass yield. The magnetic field appears to shift the metabolic balance toward biomass formation rather than ethanol production, suggesting its potential to modulate yeast metabolism (Table 1).

**Table 1:** Effect of electromagnetic fields (2.5, 10 and 15 mT) on ethanol and biomass yield under aerobic and anaerobic conditions

		Difference (%)			
		Control	2.5 mT	10 mT	15 mT
Yield _(ethanol/glucose)_	Aerobic conditions	0	− 15.1	− 28.2	− 21.9
	Anaerobic conditions	0	− 7.8	− 26.7	− 25.6
Yield _(biomass/glucose)_	Aerobic conditions	0	+ 8.7	+ 38.1	+ 70.6
	Anaerobic conditions	0	+ 23.2	+ 63.8	+ 67.6

A greater percentage of Gomem cells than did the control, and orange indicates a lower value than did the control (experimental parameters: mixing: 500 RPM, aeration with oxygen/nitrogen: 2000 cm^3^/min, pH: 5.5, temperature: 30 °C).

Magnetic field exposure to both 10 and 15 mT resulted in a faster onset of the stationary phase of growth, with a speedup of approximately 2 h under aerobic conditions and 1 h under anaerobic conditions. Faster ethanol production and glucose consumption were observed only under aerobic conditions.

To better describe the differences between aerobic and anaerobic cultivation, we evaluated the rate of biomass and ethanol production. Under both aerobic and anaerobic cultivation, the rate of biomass production was greater during the first 4 h of cultivation and subsequently decreased after the sixth hour of cultivation. The yeast exposed to the magnetic field reached the stationary phase earlier, while the yeast in the control needed more time (Table 3). The difference between aerobic and anaerobic cultivation was evident in the rate of ethanol production. In aerobic cultivation, the rate of ethanol production was greater during the first 4 h of cultivation. However, during anaerobic cultivation, the rate of ethanol production was lower than that in the control during the whole period (Table 2).

**Table 2:** Effect of electromagnetic fields (2.5, 10 and 15 mT) on the rate of ethanol and biomass production under aerobic and anaerobic conditions

		Difference compared to control (%)			
		Ethanol production		Biomass production	
	Magnetic field (mT)	Exponential phase	Stationary phase	Exponential phase	Stationary phase
Aerobic conditions	10	+ 233.3	− 118.4	+ 297.4	− 48.7
	15	+ 211.1	− 97.7	+ 352.5	− 24.6
Anaerobic conditions	10	− 5.1	− 10.5	+ 175.4	− 85.5
	15	− 10.7	− 34.2	+ 174	− 3.1

Exponential phase: first 4 h of cultivation; stationary phase: from the 4th to 9th hours of cultivation. A greater percentage of Gomem cells than did the control, and orange indicates a lower value than did the control (experimental parameters: mixing: 500 RPM, aeration with oxygen/nitrogen: 2000 cm^3^/min, pH: 5.5, temperature: 30 °C).

The conditions for ethanol production are already optimal, and after magnetic field exposure, carbon is preferably incorporated into biomass rather than into ethanol but does not speed up metabolism^19^. Under aerobic cultivation, the influence of the magnetic field is more obvious in accelerating metabolism by increasing ion and substrate transfer across the cell membrane^13^ and possibly enhancing ATP production by affecting the mitochondrial membrane, which participates in respiratory metabolism only under aerobic conditions^20^.

In our study, under both aerobic and anaerobic conditions, the dependence between the increase in magnetic field flux density and biomass production was linear, but for ethanol production, the dependence was nonlinear over most of the exposure time (Table 3).

**Table 3:** Coefficients of determination for the dependence of the electromagnetic field flux density (2.5, 10 and 15 mT) on ethanol production under aerobic and anaerobic conditions

			Exponential phase		Stationary phase		
		Time (h)	2	4	6	8	9
*R*^2^	Ethanol production	Aerobic conditions	0.8384	0.8286	0.7707	0.9187	0.0469
		Anaerobic conditions	0.3202	0.7712	0.9806	0.8882	0.5866
	Biomass production	aerobic conditions	**0.9247**	**0.9634**	**0.9898**	**0.9862**	**0.9859**
		Anaerobic conditions	**0.9329**	**0.9075**	0.8421	**0.9308**	**0.9242**

The bold cell had an *R*^2^ ≥ 0.9 (experimental parameters: mixing: 500 RPM, aeration with oxygen/nitrogen: 2000 cm^3^/min, pH: 5.5, temperature: 30 °C).

Based on the calculations of standard deviations, the stimulation effects of 10 mT and 15 mT did not differ from each other. This may be due to the maximum growth rate being reached under current conditions or the "window" effect of the magnetic field, as described by a few authors^8,13^. The “window effect phenomenon” suggests that the relationship between the strength or frequency of an electromagnetic field is nonlinear and that certain field parameters can stimulate or inhibit organisms significantly more than slightly stronger or weaker magnetic fields^13,21^.

In contrast to our results documenting the stimulating effect of magnetic fields, various authors have found either inhibitory effects^11,12,22^ or no effect of magnetic fields^23,24^. The observed inhibitory effect might be caused by the use of extremely strong magnetic fields up to 14 T, which can cause rupture of the plasmatic membrane of yeast^11^, or by the use of yeast strains defective in homologous DNA repair^12^. A positive effect of a magnetic field has been observed only for small volumes using an electromagnetic field ranging from 0.5 to 25 mT^8,25,26^ or a stronger magnetic field induced by magnets^9^.

Perez et al.^13^ were the only ones to study alcohol fermentation of S. cerevisiae with a magnetic field applied in a larger volume (5 L). All other authors investigating the impact of magnetic fields on yeast growth and fermentation worked with volumes up to 250 ml^9,24,25^.. The authors Perez et al.^13^ reported a 17% increase in biomass and ethanol production when using a stronger magnetic field (25 mT). While few studies have been devoted to this topic, when explaining the results of our work, we relied on the study of Perez et al. ^13^, who, like us, observed a stimulation of biomass production in a larger volume (5 L), although not to the same extent as in our work. In these terms, our findings can be explained by attributing these findings to the impact of the magnetic field on enhancing membrane permeability, which, in turn, influences the ion transport mechanism across the cellular membrane. In our opinion, a magnetic field can stimulate ion transport while concurrently reducing the cellular demand for ATP during biomass synthesis, which has been described at the cellular level in the past^27,28^. In our opinion, this mechanism leads to accelerated growth rates, enhanced metabolite production, increased biomass yield, and, intriguingly, lower yields of ethanol or glycerol, as evidenced by carbon balance calculations, which were observed in our study. To verify this assumption, additional experiments are needed, which will be the subject of our future research.

## Conclusion

When studying a weak magnetic field with magnetic flux densities of 2.5, 10 and 15 mT, we observed a statistically significant impact of magnetic field exposure on yeast growth and metabolism in both aerobic and anaerobic cultivations. We recorded a stimulation of biomass production ranging from 40 to 73% and a decrease in overall ethanol production ranging from 7 to 28% when exposed to magnetic fields of 10 and 15 mT. Notably, a magnetic field of 2.5 mT did not induce statistically significant changes in either aerobic or anaerobic cultivation.

Additionally, we observed faster glucose consumption, a faster decrease in dissolved oxygen (pO_2_) saturation, and greater nitrogen consumption, providing evidence of accelerated yeast metabolism due to magnetic field exposure. These processes occurred approximately 2 h earlier under aerobic cultivation but were not accelerated under anaerobic cultivation except for biomass production.

Based on published results, we believe that magnetic fields enhance membrane permeability, ion transport, and metabolic processes, thereby increase the metabolic rate in yeast cells. Additionally, the volume of the medium influences the biological effects of the magnetic field, contributing to our understanding of its impact on yeast fermentation.

The most noticeable stimulatory effect on yeast was observed with magnetic fields of 10 mT and 15 mT. Based on the standard deviation calculations and regression model analyses, there was no statistically significant difference in biomass production between 10 and 15 mT, but the 10 mT coil did not generate excessive heat, eliminating the need for intensive cooling of the system. A magnetic field strength of 10 mT holds promise for biotechnological process control in a 2 L working volume. Magnetic field exposure has the potential to accelerate yeast growth and metabolic processes in industrial applications, with careful consideration of optimal parameters. However, further research is needed to fully comprehend the underlying mechanisms and optimize the application of magnetic fields in biotechnological processes. Furthermore, the scalability of these findings to large-scale industrial settings should be thoroughly investigated.

In conclusion, this research contributes to the expanding body of knowledge regarding the impact of magnetic fields on microbial systems, offering valuable insights for potential biotechnological applications. Nevertheless, additional investigations are needed to comprehensively grasp the underlying mechanisms and enhance the utilization of magnetic fields in biotechnological processes.

## Methods

### Model organism

To investigate the effect of magnetic fields, we used commercial strain of Saccharomyces cerevisiae owned by Pakmaya a.s. (NCYC R680 from the National Collection of Yeast Cultures of United Kingdom). In the experiment, we used SOP Verduyn glucose liquid medium, which contained the following components: Dextrose 20.0 g/L, (NH_4_)_2_SO_4_ 5.0 g/L, KH_2_PO_4_ 3.0 g/L, MgSO_4_⋅7H_2_O 0.5 g/L, vitamin solution 1 mL/L (D-biotin 0.05 g/L, Ca-D-pantothenate 1.00 g/L, Nicotonic acid 1.00 g/L, Myo-inositol 25.00 g/L, Thiamine hydrochloride 1.00 g/L, Pyridoxal hydrochloride 1.00 g/L, p-aminobenzoic acid 0.20 g/L), and 1 mL/L trace elements (Na_2_EDTA 1.50 g/L, ZnSO_4_·7H_2_O 0.45 g/L, MnCl_2_·2H_2_O 0.10 g/L, CoCl_2_·6H_2_O 0.03 g/L, CuSO_4_·5H_2_O 0.03 g/L, Na_2_MoO_4_·2H_2_O 0.04 g/L, CaCl_2_·2H_2_O 0.45 g/L, FeSO_4_·7H_2_O 0.30 g/L, H_3_BO_3_ 0.10 g/L, KI 0.01 g/L), all provided by Sigma-Aldrich, St. Louis, Missouri, United States. The pH of the medium was maintained at 5.5 throughout the entire cultivation period with 20% (v/v) H2SO4 and 25% (w/v) NaOH.

### Electromagnetic field

The stimulating magnetic field was created by passing an electrical current through a specially designed circular coil immersed directly in the cultivation media for the entire exposure time (24 h). For this experiment, we constructed a copper circular coil measuring 5 cm in length and 1 cm in width. The magnetic field was measured at the center of the coil. The electromagnetic field was induced using an electromagnetic generator. The stimulation fields magnetic flux density (B) in the coil system was measured using the VEMA 04 fluxgate magnetometer, which had a sensitivity of 2 nT/LSB and a sampling frequency of 1000 Hz. The magnetometer was calibrated before the measurements using a method that utilized a neural network.

### Experimental design

We conducted separate control and experimental cultivations. The experiments took place in a 3 L fermenter with a working volume of 2 L (Sartorius, BIOSTAT® A, Germany). The coil was inserted inside and autoclaved along with the medium. The yeast cells were inoculated to achieve a dry cell concentration of 1 g/L. The cultivation was carried out under stable conditions in laboratory fermenter with exhaust cooling system, stabile pH of 5.5, a temperature of 30 °C, 500 RPM stirring, aerated with oxygen for aerobic cultivation and the present oxygen has swept with nitrogen for anaerobic cultivation at a rate of 2000 cm^3^/min. Throughout the experiment, online data such as exhaust O_2_, exhaust CO_2_, the volumes of base or acid used to maintain stable pH, and temperature were recorded.

The experimental cultures were exposed to magnetic fields for 24 h of magnetic flux density of 2.5 mT, 10 mT, or 15 mT, while the control cultures were maintained under laboratory conditions for the entire 24-h duration of the experiment.

During the entire cultivation period, we withdrew 5 ml of cell culture using a sterile syringe every hour (excluding the nighttime) to measure the optical density (OD) at 600 nm and to prepare samples for HPLC (Agilent Technologies) by filtering the samples via Sartorius RC 0.45 µm filters. HPLC analysis was performed with Shodex SUGAR SH10118 × 300 mm column, 6 µm, at 50 °C, 0.005 M H2SO4 as mobile phase at 0.800 mL/min flow rate and the metabolites detected with a refractive index detector (RID). Ammonium nitrogen was measured via semi-automated Kjeldahl analyzer (BUCHI, Kjelflex K-360, Switzerland).

The data were statistically evaluated using online tool (https://www.socscistatistics.com) and the Minitab statistical software 22.1.0 (https://www.minitab.com/en-us/). To analyze biomass and ethanol production the regression analysis was chosen as the most suitable statistical method.

### Measured parameters

The optical density (OD) at 600 nm was measured using a UV–vis spectrophotometer (Shimadzu, UV-1900i, Japan). Each cell culture was diluted with distilled water to obtain OD values within the range of 0.02 to 0.9. Samples for HPLC analysis were prepared by first filtering the cell samples using a cell filter with pore sizes of 45 µm into new microtubes. These samples were then stored in a refrigerator. Just before the HPLC analysis, the samples were diluted 10 × by adding 100 µl of the filtered sample to 900 µl of ultra-pure distilled water. We analyzed all samples together after each experiment. We used HPLC to measure the concentrations (g/L) of sugars (glucose, maltose), ethanol, glycerol. The remaining undiluted filtered sample was used for ammonium nitrogen measurement using the similar method.

In aerobic cultivation, we also measured exhaust gas CO_2_ amount as percentile (Sartorius, BioPAT Xgas, Germany) and calculated the carbon mole accordingly to introduced air volume. Carbon mole in the exhaust gas and carbon mole of produced biomass, ethanol and consumed sugars are used to perform the carbon balance. The CO_2_ measurement could only be performed in aerobic cultivation since the analyser was not adapted to measure high concentrations of CO_2_ without the presence of oxygen, as seen during anaerobic cultivation.

### Supplementary Information

Supplementary Information 1.

Supplementary Information 2.

Supplementary Information 3.

Supplementary Information 4.

Supplementary Information 5.

## Data Availability

The datasets used and/or analysed during the current study available from the corresponding author on reasonable request.

## References

[1] Chen GQ, Jiang XR (2018). Next generation industrial biotechnology based on extremophilic bacteria. Curr. Opin. Biotechnol..

[2] Sedlakova-Kadukova J (2022). Microorganisms in metal recovery—Tools or teachers?. Microb. Syntrophy-Mediat. Eco-enterpris. Acad Press.

[3] Sincak M, Luptakova A, Matusikova I, Jandacka P, Sedlakova-Kadukova J (2023). Application of a magnetic field to enhance the environmental sustainability and efficiency of microbial and plant biotechnological processes. Sustainability.

[4] Dabros M, Schuler MM, Marison IW (2010). Simple control of specific growth rate in biotechnological fed-batch processes based on enhanced online measurements of biomass. Bioprocess Biosyst. Eng..

[5] Saliev T, Begimbetova D, Masoud AR (2016). Biological effects of non-ionizing electromagnetic fields: Two sides of a coin. Prog. Biophys. Mol. Biol..

[6] Sotirios-Spyridon V, Kapelos J (2020). Factors affecting yeast ethanol tolerance and fermentation efficiency. World J. Microbiol. Biotechnol..

[7] Hocalar A, Türker M, Karakuzu C, Yüzgeç U (2011). Comparison of different estimation techniques for biomass concentration in large scale yeast fermentation. ISA Transact..

[8] Mehedintu M, Berg H (1997). Proliferation response of yeast Saccharomyces cerevisiae on electromagnetic field parameters. Bioelectrochem. Bioenerg..

[9] da Motta MA, Muniz JBF, Schuler A, Da Motta M (2004). Static magnetic fields enhancement of Saccharomyces cerevisae ethanolic fermentation. Biotechnol Prog..

[10] Novák J, Strašák L, Fojt L, Slaninová I, Vetterl V (2007). Effects of low-frequency magnetic fields on the viability of yeast Saccharomyces cerevisiae. Bioelectrochemistry.

[11] Iwasaka M, Ikehata M, Miyakoshi J, Ueno S (2004). Strong static magnetic field effects on yeast proliferation and distribution. Bioelectrochemistry.

[12] Ruiz-Gómez MJ, Sendra-Portero F, Martínez-Morillo M (2010). Effect of 2.45 mT sinusoidal 50 Hz magnetic field on Saccharomyces cerevisiae strains deficient in DNA strand breaks repair. Int. J. Radiat. Biol..

[13] Perez VH, Reyes AF, Justo OR, Alvarez DC, Alegre RM (2007). Bioreactor coupled with electromagnetic field generator: effects of extremely low frequency electromagnetic fields on ethanol production by saccharomyces cerevisiae. Biotechnol. Prog..

[14] Binhi VN, Prato FS (2016). A physical mechanism of magnetoreception: extension and analysis. Bioelectromagnetics.

[15] Brice C, Cubillos FA, Dequin S, Camarasa C, Martínez C (2018). Adaptability of the Saccharomyces cerevisiae yeasts to wine fermentation conditions relies on their strong ability to consume nitrogen. PLoS ONE.

[16] Gorte O, Kugel M, Ochsenreither K (2020). Optimization of carbon source efficiency for lipid production with the oleaginous yeast Saitozyma podzolica DSM 27192 applying automated continuous feeding. Biotechnol. Biofuels..

[17] Mutton MJR, Ferrari FC, Freita LAD (2019). Interaction between the production of ethanol and glycerol in fed-batch bioreactors. Braz. J. Microbiol..

[18] González-Hernández Y, Michiels E, Perré PA (2022). Comprehensive mechanistic yeast model able to switch metabolism according to growth conditions. Fermentation.

[19] Halász A., Lásztity R. Use of yeast biomass in food production. *Routledge*. (2017)

[20] Ogneva IV, Usik MA, Burtseva MV, Biryukov NS, Zhdankina YS, Sychev VN, Orlov OI (2020). Drosophila melanogaster sperm under simulated microgravity and a hypomagnetic field: Motility and cell respiration. Int. J. Mol. Sci..

[21] Binhi VN, Prato FS (2017). Biological effects of the hypomagnetic field: An analytical review of experiments and theories. PLoS ONE.

[22] Ruiz-Gómez MJ, Prieto-Barcia MI, Ristori-Bogajo E, Martınez-Morillo M (2004). Static and 50 Hz magnetic fields of 035 and 245 mT have no effect on the growth of Saccharomyces cerevisiae. Bioelectrochemistry.

[23] Kladko DV, Zakharzhevskii MA, Vinogradov VV (2020). Magnetic field-mediated control of whole-cell biocatalysis. J. Phys. Chem. Lett..

[24] Boeira CZ, de Carvalho Silvello MA, Remedi RD, Feltrin ACP, Santos LO, Garda-Buffon J (2021). Mitigation of nivalenol using alcoholic fermentation and magnetic field application. Food Chem..

[25] Santos LO, Alegre RM, Garcia-Diego C, Cuellar J (2010). Effects of magnetic fields on biomass and glutathione production by the yeast Saccharomyces cerevisiae. Process Biochem..

[26] Canli O, Kurbanoğlu EB (2012). Application of low magnetic field on inulinase production by Geotrichum candidum under solid state fermentation using leek as substrate. Toxicol. Ind. Health.

[27] Buchachenko AL, Kuznetsov DA (2008). Magnetic field affects enzymatic ATP synthesis. J. Am. Chem. Soc..

[28] Zhao G (2011). Cellular ATP content was decreased by a homogeneous 85 T static magnetic field exposure: role of reactive oxygen species. Bioelectromagnetics.

