# Supplementary figures and images for: Exploring the impact of magnetic fields on biomass production efficiency under aerobic and anaerobic batch fermentation of Saccharomyces cerevisiae
Source: Sci Rep. 2024 Jun 4;14:12869. doi: 10.1038/s41598-024-63628-1 (PMC11150259; doi:10.1038/s41598-024-63628-1)

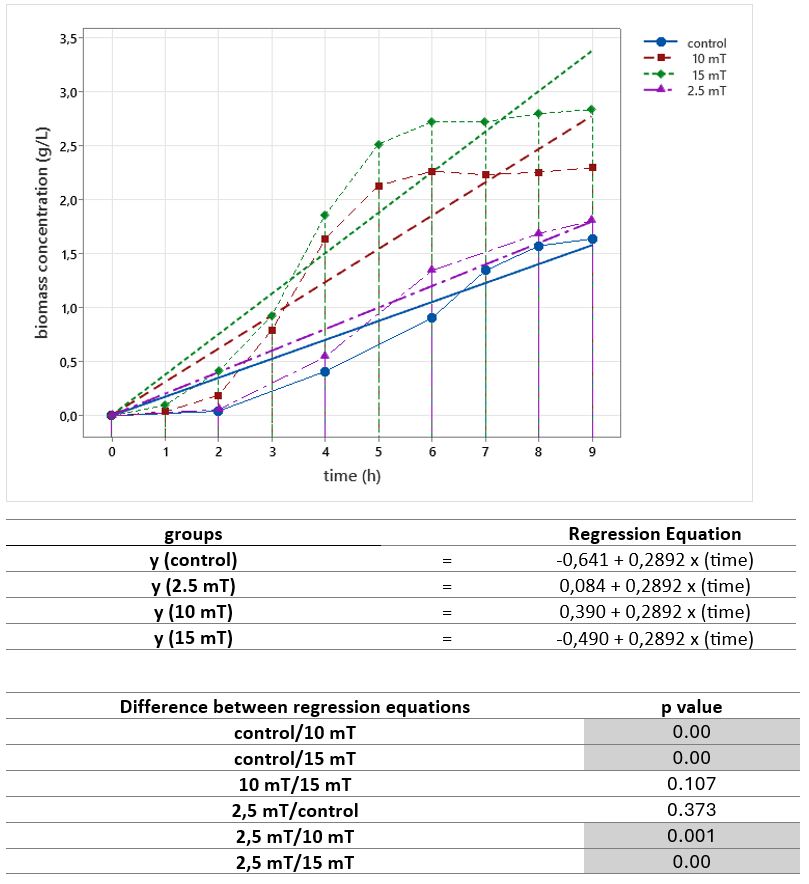

Supplement: Supplementary file 1 — Supplementary Information 1. [file 41598_2024_63628_MOESM1_ESM.jpg]

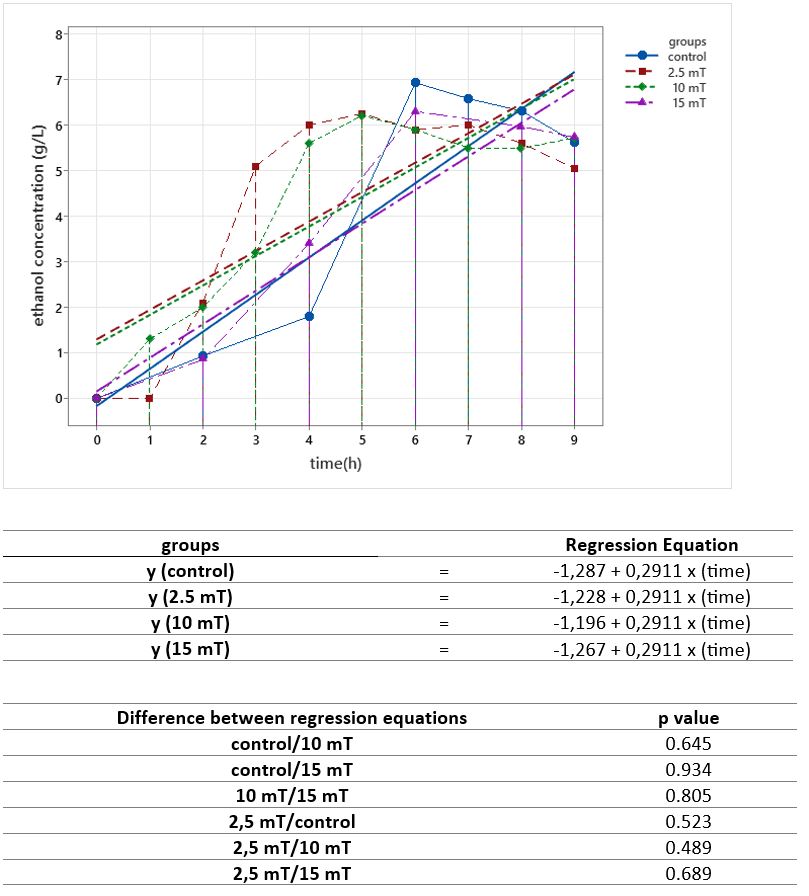

Supplement: Supplementary file 2 — Supplementary Information 2. [file 41598_2024_63628_MOESM2_ESM.jpg]

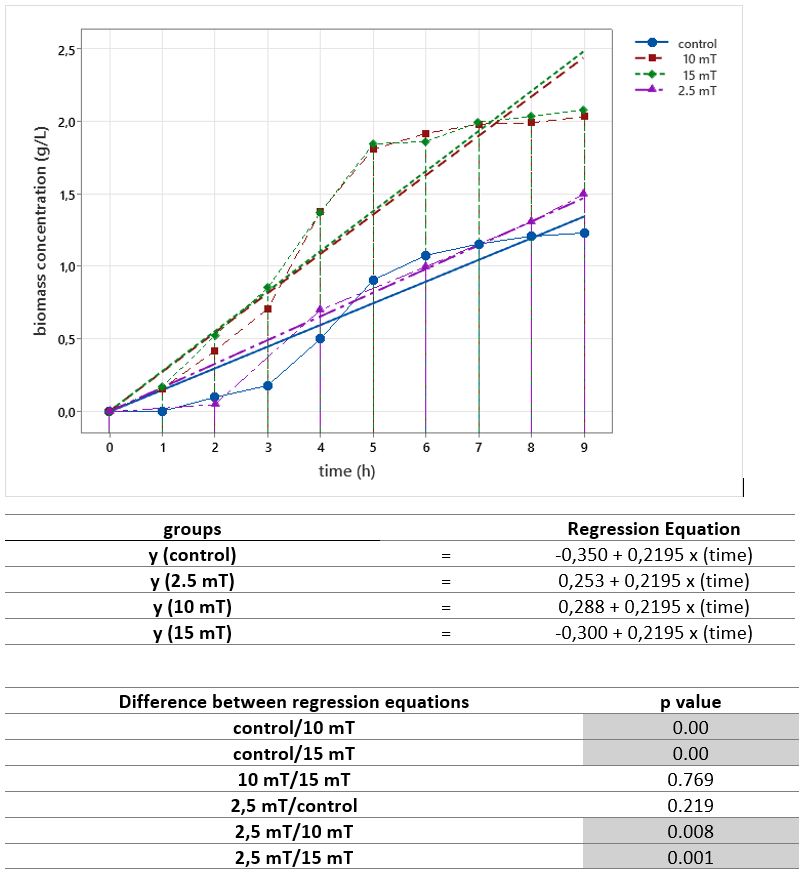

Supplement: Supplementary file 3 — Supplementary Information 3. [file 41598_2024_63628_MOESM3_ESM.jpg]

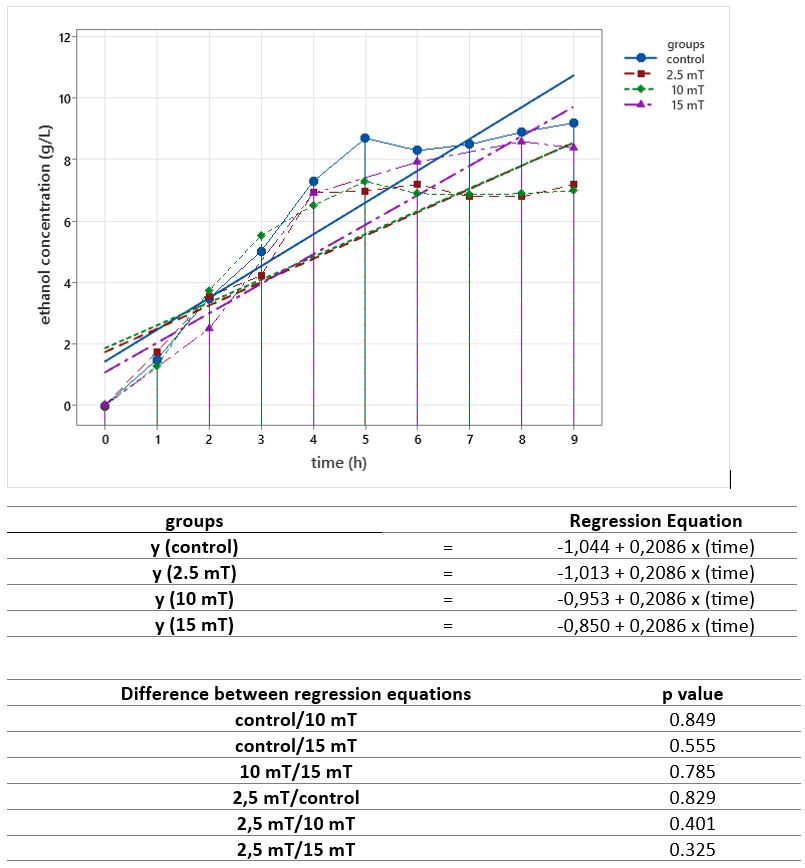

Supplement: Supplementary file 4 — Supplementary Information 4. [file 41598_2024_63628_MOESM4_ESM.jpg]
