# Supplementary material for: Exploring the impact of magnetic fields on biomass production efficiency under aerobic and anaerobic batch fermentation of Saccharomyces cerevisiae
Source: Sci Rep. 2024 Jun 4;14:12869. doi: 10.1038/s41598-024-63628-1 (PMC11150259; doi:10.1038/s41598-024-63628-1)
Supplement: Supplementary file 5 — Supplementary Information 5. [file 41598_2024_63628_MOESM5_ESM.pdf]

Exploring the impact of magnetic fields on biomass production efficiency under aerobic and anaerobic batch fermentation of Saccharomyces cerevisiae.

M. Sincak[1], M. Turker[2], Ü. C. Derman[2], A. Erdem[2], P. Jandacka[3], M. Luptak[4], A. Luptakova[5], J. Sedlakova-Kadukova[1,6]*

[1]University of Ss. Cyril and Methodius in Trnava, Faculty of Natural Science, Nam. J. Herdu 2, 917 01 Trnava, Slovakia

[2]Pak Gida Uretim Ve Paz. A.S., Kartepe, Kocaeli, Turkey

[3]Czech University of Life Sciences Prague, Faculty of Forestry and Wood Sciences, Kamycka 129, 16500 Praha 6 – Suchdol, Czech Republic

[4]Technical University of Kosice, Faculty of Materials, Metallurgy and Recycling, Letna 9, 04200 Kosice, Slovakia;

[5]Slovak Academy of Sciences, Institute of Geotechnics, Watsonova 45, 04001 Kosice, Slovakia

[6]ALGAJAS s.r.o., Prazská 16, 04011 Kosice, Slovakia

*Correspondence: e-mail address: jana.sedlakova.fpv@ucm.sk, jana.sedlakova@algajas.com

+these authors contributed equally to this work

Supplementary material

Figure S1 The regression analyses of biomass production in aerobic cultivation after the magnetic field exposure (2.5, 10 and 15 mT) compared to the control (in laboratory conditions). The shaded cell indicates significant change according to regression analyses ($p \geq 0.05$)

Figure S2 The regression analyses of ethanol production in aerobic cultivation after the magnetic field exposure (10 and 15 mT) compared to the control (in laboratory conditions). The shaded cell indicates significant change according to regression analyses ($p \geq 0.05$)

Figure S3 The regression analyses of biomass production in anaerobic cultivation after the magnetic field exposure (2.5, 10 and 15 mT) compared to the control (in laboratory conditions). The shaded cell indicates significant change according to regression analyses ($p \geq 0.05$)

Figure S4 The regression analyses of ethanol production in anaerobic cultivation after the magnetic field exposure (10 and 15 mT) compared to the control (in laboratory conditions). The shaded cell indicates significant change according to regression analyses ($p \geq 0.05$)
